# *In Situ* Conversion of Melanoma Lesions into Autologous Vaccine by Intratumoral Injections of α-gal Glycolipids

**DOI:** 10.3390/cancers2020773

**Published:** 2010-05-04

**Authors:** Uri Galili, Mark R. Albertini, Paul M. Sondel, Kim Wigglesworth, Mary Sullivan, Giles F. Whalen

**Affiliations:** 1 Department of Surgery, University of Massachusetts Medical School, 55 Lake Avenue North, Worcester, MA 01655, USA; E-Mails: kim.wigglesworth@umassmed.edu (K.W.); Mary.Sullivan3@umassmemorial.org (M.S.); Giles.Whalen@umassmemorial.org (G.F.W.); 2 University of Wisconsin Carbone Cancer Center, Madison, WI. 53792, USA; E-Mails: mralbert@wisc.edu (M.R.A.); pmsondel@humonc.wisc.edu (P.M.S.)

**Keywords:** Melanoma immunotherapy, anti-Gal antibody, α-gal glycolipids, α1,3-galactosyltransferase, Fcγ receptors, antigen presenting cells

## Abstract

Autologous melanoma associated antigens (MAA) on murine melanoma cells can elicit a protective anti-tumor immune response following a variety of vaccine strategies. Most require effective uptake by antigen presenting cells (APC). APC transport and process internalized MAA for activation of anti-tumor T cells. One potential problem with clinical melanoma vaccines against autologous tumors may be that often tumor cells do not express surface markers that label them for uptake by APC. Effective uptake of melanoma cells by APC might be achieved by exploiting the natural anti-Gal antibody which constitutes ~1% of immunoglobulins in humans. This approach has been developed in a syngeneic mouse model using mice capable of producing anti-Gal. Anti-Gal binds specifically to α-gal epitopes (Galα1-3Galβ1-4GlcNAc-R). Injection of glycolipids carrying α-gal epitopes (α-gal glycolipids) into melanoma lesions results in glycolipid insertion into melanoma cell membranes, expression of α-gal epitopes on the tumor cells and binding of anti-Gal to these epitopes. Interaction between the Fc portions of bound anti-Gal and Fcγ receptors on APC induces effective uptake of tumor cells by APC. The resulting anti-MAA immune response can be potent enough to destroy distant micrometastases. A clinical trial is now open testing effects of intratumoral α-gal glycolipid injections in melanoma patients.

## 1. Why Autologous Tumor Vaccines in Melanoma Patients

In many melanoma patients, appearance of metastases that are refractory to therapy is usually lethal. Destruction of such metastases by resection or ablation provides only a temporary solution, since residual micrometastases most often continue to develop into lethal lesions. One potential approach to destroy residual micrometastases may be the use of immunotherapy, *i.e.*, stimulation of the immune system to detect and destroy tumor cells expressing melanoma associated antigens (MAA). Much has been learned about the mechanisms involved in a variety of immunotherapy approaches by studying anti-tumor immune reactions in tumor-bearing mice. Careful evaluation of *in vitro* responses of melanoma patients, particularly those receiving experimental forms of immunotherapy, have provided insights regarding human MAA and components of the immune response involved in their recognition. Some MAA may be shared by tumors in various patients (e.g., MAGE, Mart-1, tyrosinase, gp75, gp100), but the extent of their expression varies between individual patients [1,2]. Additional MAA are likely to be specific to the patient and are designated as autologous, unique, or private MAA. These autologous, private MAA are generated by the multiple mutations resulting from the genomic instability of tumor cells. Many of these mutations in cancer cells are unique to the individual patient and result in mutated proteins that provide growth advantage to the tumor cells [3,4]. Other mutations are neutral since they do not affect the structure or function of the mutated protein. Nevertheless, most mutated proteins may serve as targets for immunotherapy that elicits a specific immune response against tumor cells. Such an immune response is based on the fact that these mutated proteins expressing autologous MAA are “foreign antigens” to the patient; namely, they are not present in the normal cells of the individual patient. Absence of such autologous MAA on normal cells enables the immune system to differentiate between normal cells and the malignant cells.

The assumption that the immune system in humans is capable of detecting and destroying tumor cells carrying autologous tumor antigens is based primarily on retrospective studies demonstrating a distinct correlation between the extent of T cell infiltration into tumors inspected post resection, and prognosis in the individual patient [5,6,7,8]. These reported correlations imply that the immune system in some patients is capable of developing a specific protective response against antigens that are unique to the tumor cells. This protective process may destroy metastatic tumor cells and improve prognosis in that individual patient. The retrospective histological analyses suggest that effective cancer immunotherapy may be achieved by eliciting an immune response against tumor antigens within the individual patient. Studies in lymphoma patients immunized against the immunoglobulin isotype expressed on their tumor cells (*i.e.*, a unique autologous tumor antigen) indicated that after initial destruction of many tumor cells, the tumor evades the elicited immune response by selective development of tumor cells that lack the isotype [9,10]. This implies that effective immunotherapy may require the induction of an immune response against multiple antigens specific to the tumors cells in the treated patient in order to prevent escape from anti-tumor immunity via the loss of a sole tumor antigen. Currently, it is impossible to identify the full range of autologous tumor antigens in each individual cancer patient in order to generate a vaccine of purified antigens or peptides that reflect the spectrum of autologous MAA on that patient’s melanoma. Nevertheless, even without the ability to characterize the individual MAA, a patient’s autologous tumor may be a source of the spectrum of autologous MAA. For this reason, a number of strategies have considered using a patient’s autologous tumor cells as a possible source of vaccinating tumor antigens, provided that these antigens can be presented to the immune system in an immunogenic form.

## 2. Immunogenicity of Autologous Tumor Antigens May be Increased by Fc/FcγR Targeting to Antigen Presenting Cells (APC)

Antigens that are unique to the tumor may be expressed on the tumor cells also in advanced stages of the disease. However, the immune system in these patients is often severely suppressed by diverse mechanisms. For this reason, the immune systems of patients with rapidly advancing cancers may be “oblivious” to the presence of the MAA, and is not effectively activated by the tumor to induce a protective MAA-specific immune response. A common reason for such a lack of immune response to tumor antigens seems to be failure of antigen presenting cells (APC) to detect tumor cells, in order to internalize them and then to effectively process and present their immunogenic tumor antigen peptides. In order to elicit a protective anti-tumor immune response, APC such as dendritic cells and macrophages have to internalize the tumor cells, transport them to the draining lymph nodes, and process the tumor antigens into peptides that are presented by MHC class I and class II molecules for the activation of tumor specific CD8+ and CD4+ T cells, respectively [11,12,13]. Once these tumor specific T cells are activated, they proliferate, leave the lymph node and circulate in the body in order to seek and destroy metastatic tumor cells that express the tumor antigens. In the setting of advanced disease, tumor cells can evolve by a process analogous to “natural selection”. Due to this selective process their phenotype can be modified so that they lack any marker identifying them as cells that should be recognized and internalized by APC, thereby avoiding the development of a protective anti-tumor immune response [1,14]. This implies that if tumor cells can be targeted to APC for effective internalization, their tumor antigens are likely to be processed by the APC and presented for the effective activation of tumor specific T cells. Such targeting can be achieved by coating tumor cells with an antibody followed by interaction of the Fc portion of the antibody with Fcγ receptors (FcγR) on APC.

Both dendritic cells and macrophages have on their cell membranes FcγR that can bind the Fc portion of IgG antibodies following the formation of immune complexes between such antibodies and their corresponding antigens [15,16,17]. This Fc/FcγR interaction results in a much more effective uptake and internalization of immunocomplexed vaccines than internalization of vaccines that are not immunocomplexed. Because of this targeting to APC via Fc/FcγR interaction, immunization with various vaccines that are immunocomplexed with antibodies elicit a more effective immune response against viruses, bacteria or protozoa than immunization with the same microbial agents that are not immunocomplexed [18,19,20,21,22,23,24]. Similarly, tumor cells coated with an anti-tumor antibody were reported to undergo effective uptake by APC and subsequent effective processing and presentation of the internalized tumor antigens by these APC, whereas internalization by APC of tumor cells lacking coating immunoglobulins was found to be poor [25,26,27].

The “catch” in this method for increasing vaccine immunogenicity by targeting via Fc/FcγR interaction is the requirement for an antibody that will form immune complexes with the autologous tumor within individuals that usually lack such antibodies prior to immunization. The approach we are pursuing is attempting to overcome this deficiency by exploiting the most abundant natural antibody in humans, the natural anti-Gal antibody. Our preclinical data document that this natural anti-Gal antibody can interact with α-gal epitopes expressed on tumor cells within lesions that are injected with α-gal glycolipids, induce their destruction and further induce an active anti-tumor immune response. 

## 3. The Natural Anti-Gal Antibody and Its Ligand the α-gal Epitope

**Figure 1:**
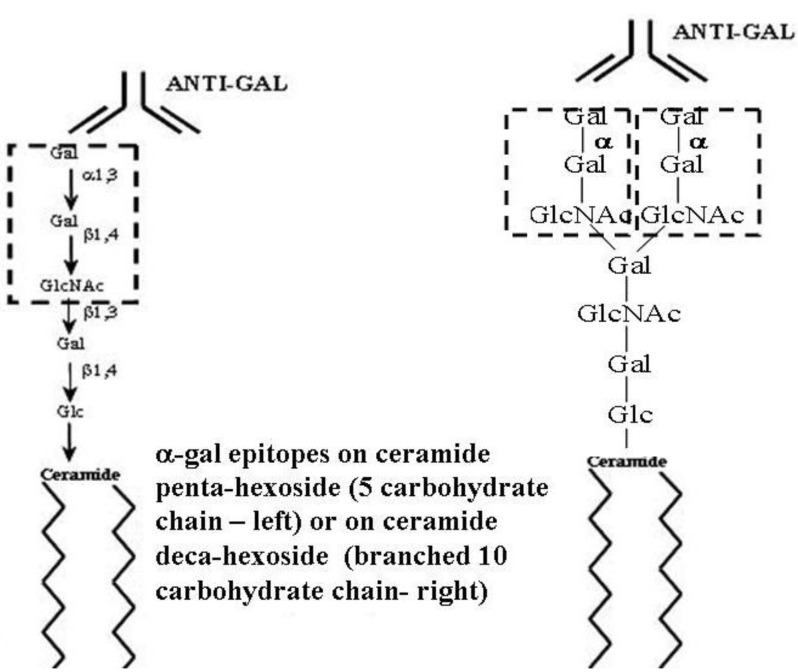
Ceramide pentahexoside (CPH) (left) and ceramide decahexoside (CDecaH) (right) as representative α-gal glycolipids. CPH is the most abundant glycolipid in rabbit RBC and is presented as a schematic α-gal glycolipid with five carbohydrates. CDecaH is a glycolipid with 10 carbohydrate branched chain. α-gal epitopes (Galα1-3Galβ1-4GlcNAc-R) are marked by the broken line rectangles. The terminal α-galactosyl (Gal) is linked α1,3 to the penultimate Gal of the carbohydrate chain by the glycosylation enzyme α1,3galactosyltransferase (α1,3GT). The carbohydrate chain is linked to the lipid portion (ceramide) embedded in the cell membrane via the two fatty acid tails. Anti-Gal binding to α-gal epitopes is presented as schematic IgG molecules. α-gal glycolipids in rabbit RBC (with the exception of ceramide heptahexoside) increase in size in increments of five carbohydrates, each forming an additional branch that is capped by α-gal epitopes.

Anti-Gal is the most abundant natural antibody in human serum, constituting ~1% of serum IgG [28]. It is produced as a result of continuous antigenic stimulation by bacteria of the gastrointestinal flora [29]. Anti-Gal interacts specifically with a carbohydrate antigen called the α-gal epitope that has the structure Galα1-3Galβ1-4GlcNAc-R [30,31]. The α-gal epitope is present on cell surface glycolipids and glycoproteins of nonprimate marsupial and placental mammals, in prosimians and New World monkeys (monkeys of South America), [32,33,34]. With the exception of spermatozoa, all cells tested in these species were found to express many α-gal epitopes [32,33,34,35]. The α-gal epitope on mammalian glycolipids is illustrated in Figure 1. α-gal epitopes are synthesized on carbohydrate chains of glycolipids and glycoproteins in the Golgi apparatus of mammalian cells by the glycosylation enzyme α1,3galactosyltransferase (α1,3GT) [33,35]. All mammals producing α-gal epitopes lack the natural anti-Gal antibody, whereas, humans, apes, and Old World monkeys (monkeys of Asia and Africa) lack α-gal epitopes due to inactivation of the α1,3GT gene in ancestral primates, but they all produce the natural anti-Gal antibody [32,33,34,35,36].

Since anti-Gal is present in all immunocompetent humans and Old World monkeys, administration of α-gal epitopes will always result in formation of immune complexes with this antibody. One area demonstrating this antigen/antibody interaction has been xenotransplantation, in which pig cells or pig organs are transplanted into humans or monkeys. Binding of the natural anti-Gal antibody to the multiple α-gal epitopes on cells of pig xenografts causes the rapid rejection of xenografts (e.g., pig heart or kidney) in humans, or in monkeys, by complement dependent cytotoxicity (CDC) and by antibody dependent cell cytotoxicity (ADCC) of endothelial cells followed by rapid collapse of the vascular bed [37,38,39,40]. Our preclinical data in tumor bearing mice (see below), imply that the injection of α-gal glycolipids into melanoma lesions results in expression of α-gal epitopes on the melanoma cells within the treated lesion, in a manner similar to the expression of these epitopes on pig cells. Provided that anti-Gal antibody is present, the subsequent binding of this antibody to the α-gal epitopes *de novo* expressed on the tumor cells results in destruction of the treated lesion, similar to xenograft rejection. Bound anti-Gal further targets the tumor cells for uptake and internalization by APC via Fc/FcγR interaction, leading to the subsequent effective processing and presentation of the tumor antigens by APC (see below). 

Studies on dendritic cells functioning as APC have indicated that the Fc/FcγR interaction is a highly effective mechanism by which these APC internalize antigens for subsequent stimulation of the immune system, since this interaction generates signals for antigen internalization as well as for maturation of dendritic cells internalizing the antigen [13,17,23,25,26,27]. Macrophages also express FcγR that enable them to internalize tumor cells coated with an IgG antibody, such as anti-Gal. This uptake of opsonized tumor cells by APC results in effective transport, processing and presentation of the autologous TAA peptides for activation of TAA specific T cells in the draining lymph nodes [25,26,27]. Since the natural anti-Gal antibody is present in all immunocompetent humans, it may be exploited to serve as a universal antibody for targeting vaccinating tumor cells to APC, provided that the tumor cells are manipulated to express α-gal epitopes [41,42].

## 4. Increased Immunogenicity of Vaccines Immunocomplexed with the Anti-Gal Antibody

The immunogenicity of vaccines can be substantially augmented by forming immune complexes with anti-Gal. The extent of increased immunogenicity of such vaccines due to anti-Gal mediated targeting for uptake by APC, could be demonstrated with viral vaccines. Since mouse strains usually used for vaccine studies express α-gal epitopes on most of their cells (like other nonprimate mammals), they are not suitable for studies involving endogenous anti-Gal. Because the α-gal epitope is an autologous antigen in mice, mice are immunotolerant to it and can not produce the anti-Gal antibody. Anti-Gal can be produced, however, in knockout mice for the α1,3GT gene (referred to as KO mice). These mice lack the α-gal epitope because of targeted disruption of the α1,3GT gene [43]. In the absence of α-gal epitope, the mice are not immunotolerant to it and are capable of producing the anti-Gal antibody. Since KO mice are kept within a sterile environment, they lack the normal gastrointestinal bacteria that express α-gal epitopes and thereby elicit natural anti-Gal antibody production. However, KO mice can be induced to produce anti-Gal in titers comparable to those in humans by immunization with xenogeneic cells or cell membranes expressing α-gal epitopes. Homogenates of pig kidney membranes (PKM) express an abundance of α-gal epitopes and were found to serve as a convenient immunogen for inducing anti-Gal responses in KO mice [44,45].

Increased immunogenicity of viral vaccines targeted to APC by anti-Gal was first studied in KO mice that were immunized with the envelope glycoprotein gp120 of HIV, or with the same glycoprotein which was modified to also express multiple α-gal epitopes. Expression of the α-gal epitope on the latter vaccine was achieved by the use of recombinant α1,3GT [46]. Both T cell response and antibody responses to the immunizing gp120 that has α-gal epitopes were found to be ~100-fold higher than those following immunization with gp120 that lacked α-gal epitopes [44]. Similarly, immunization of KO mice with inactivated influenza virus that was processed to express multiple α-gal epitopes resulted in ~100-fold higher T cell and antibody response against influenza virus than the response in KO mice immunized with inactivated influenza virus lacking α-gal epitopes [45]. Moreover, challenge of both groups of immunized mice with live influenza virus resulted in protection and survival of ~90% of mice immunized with α-gal epitope expressing influenza vaccine, whereas only ~15% of mice immunized with vaccine lacking α-gal epitopes did not die following this challenge [45]. These findings suggest that the formation of immune complexes at the vaccination site between HIV or influenza vaccines and anti-Gal, results in a much more effective uptake, processing and presentation of the immunogenic peptides within the vaccine than with similar vaccines that lack α-gal epitopes.

In order to study the mechanisms involved in anti-Gal mediated increased immunogenicity of vaccines, the immune response to hen egg ovalbumin (OVA) was analyzed in KO mice. Anti-Gal mediated targeting of OVA to APC was achieved by encapsulating OVA within liposomes that express multiple α-gal epitopes (referred to as α-gal liposomes) [47]. OVA was chosen as a model antigen since several highly sensitive immunological tools are available for measuring the processing of this antigen in APC and presentation of its most immunogenic peptide SIINFEKL on class I MHC molecules. Therefore, it is possible to evaluate the activation of CD8+ T cells following the specific interaction with SIINFEKL when presented on class I MHC molecules. Uptake and processing of OVA encapsulated in α-gal liposomes by KO mouse APC was several fold higher when the liposomes were coated with anti-Gal, than when the α-gal liposomes were not coated by this antibody, due to Fc/FcγR interaction between the Fc portion of anti-Gal on liposomes and FcγR on APC [47]. The APC mediated transport of OVA from the immunization site to draining lymph nodes, the subsequent activation of SIINFEKL specific T cells and the titer of anti-OVA antibodies, all were much higher if the vaccinated mice produced the anti-Gal antibody, than in mice lacking this antibody (*i.e.*, wild type [WT] mice) [47]. Overall, the studies with OVA encapsulated in α-gal liposomes confirmed the hypothesis that formation of immune complexes between circulating anti-Gal and α-gal epitopes on vaccines targets vaccines for increased uptake, processing, presentation by APC, as well as increased transport to draining lymph nodes [47]. The increased processing and presentation of the immunogenic vaccine peptides results in a much higher activation of CD8+ and CD4+ T cells specific to the vaccine than the immune response to the same vaccine which lacks α-gal epitopes [47]. These observations suggested that if tumor cells in cancer patients could be induced to express α-gal epitopes, they may be converted into effective autologous endogenous vaccines that are targeted by anti-Gal to APC. The next step was to determine whether α-gal epitopes could be expressed on tumor cells that were initially negative for these epitopes by direct injection of α-gal glycolipids into tumors. 

## 5. *In Situ* Expression of α-gal Epitopes on Melanoma Cells by Intratumoral Injection of α-gal Glycolipids

As indicated above, human tumor cells and normal human cells do not express α-gal epitopes because the α1,3GT gene is inactivated in humans. Previous studies with tumor cells lacking α-gal epitopes demonstrated effective synthesis of α-gal epitopes on tumor cells by *in vitro* incubation with recombinant α1,3GT [41,48], or by *in vitro* transduction with viral vectors containing the α1,3GT gene [49,50]. The efficacy of these methods in inducing *in vivo* expression of α-gal epitopes could be evaluated in B16 melanoma tumors developing in KO mice since B16 melanoma cells lack active α1,3GT and thus, lack α-gal epitopes on their cell membrane [48,49,50,51]. In contrast to the effective *in vitro* transduction, intratumoral injections of recombinant α1,3GT, or of adenovirus containing the α1,3GT gene, were found to be inefficient. These injections of the α1,3GT enzyme or the gene that encodes it did not induce the expression of significant amounts of α-gal epitopes on tumor cells within the B16 lesions, beyond the area bordering the injection site. In contrast, intratumoral injection of α-gal glycolipids was found to result in effective *in situ* expression of α-gal epitopes on the tumor cells and thus, effective induction of a protective anti-melanoma immune response [52,53].

α-gal glycolipids are glycolipids comprised of a ceramide lipid tail and a linear or branched carbohydrate chain capped with α-gal epitopes (Figure 1). Rabbit red blood cells (RabRBC) are the most convenient and richest source of α-gal glycolipids in mammals [54,55,56,57,58,59]. The shortest α-gal glycolipid in RabRBC has five carbohydrate units and is called ceramide pentahexoside (CPH) (Figure 1). The next in size is ceramide heptahexoside (CHH), which is identical in structure to CPH, with the exception that the carbohydrate chain is longer by two carbohydrate units (lactosamine Galβ1-4GlcNAc). Larger α-gal glycolipids increase in size by increments of five carbohydrates, each increment forms a new branch of the carbohydrate chain to generate glycolipids with 10, 15, 20, 25 and up to 40 carbohydrate units, in which each branch is capped with α-gal epitopes [54,55,56,57,58,59]. Most carbohydrate chains in RabRBC glycolipids are capped with α-gal epitopes (with the exception of a glycolipid with three carbohydrate chains, ceramide trihexoside [CTH] Galα1-4Galβ1-4Glc-ceramide, which is present also in human RBC) [60]. 

α-gal glycolipids are extracted from RabRBC membranes by overnight incubation in a solution consisting of chloroform and methanol. This results in the extraction of glycolipids, phospholipids and cholesterol, whereas proteins are denatured and form a precipitate that is removed by filtration [52]. Gradual addition of water to the chloroform: methanol solution results in partition into an upper aqueous phase containing mostly methanol and water and a lower organic phase containing mostly chloroform and methanol [52]. The α-gal glycolipids preferentially dissolve in the upper aqueous phase because of their hydrophilic carbohydrate chains. In contrast, phospholipids and cholesterol are much more hydrophobic and thus, dissolve within the organic phase. This partition step enables the isolation of α-gal glycolipids from the extracts of RabRBC membranes and removal of phospholipids and cholesterol. The extracted α-gal glycolipids are concentrated in a rotary evaporator and dissolved in water in the form of micelles.

**Figure 2:**
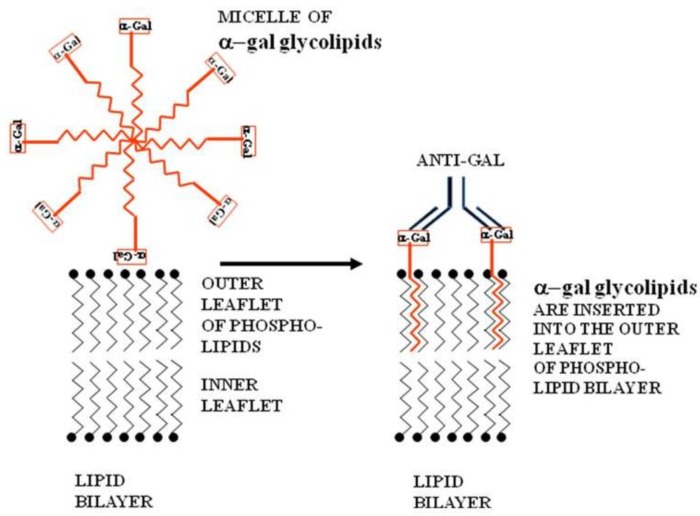
Insertion of α-gal glycolipids into the lipid bilayer of tumor cell membranes. α-gal glycolipid molecules dissolve in water or saline as micelles (ball like structures with the cross-section described in this figure) in which the hydrophobic (lipophilic) ceramide chains are clustered in the core. When these micelles are adjacent to cells, individual α-gal glycolipid molecules “jump” into the outer leaflet of the cell lipid bilayer, because the energetic state of the ceramide tail surrounded by phospholipids is much more stable than in micelles surrounded by water. α-gal glycolipid insertion into the tumor cell membrane results in expression of α-gal epitopes on the cell. Binding of the natural anti-Gal antibody to these epitopes leads to destruction of tumor cells and their uptake by APC.

The micelles of α-gal glycolipids are submicroscopic spherical structures in which the inner part is comprised of the hydrophobic fatty acid tails of the ceramide. The outer part the micelle is formed by the hydrophilic carbohydrate chains (Figure 2). When such micelles are brought near tumor cells, the α-gal glycolipid molecules “jump” into the cell membrane and the ceramide portion inserts into it. Therefore, the carbohydrate chains carrying α-gal epitopes protrudes out of the cell membrane. This insertion occurs spontaneously because the hydrophobic lipid tails of the α-gal glycolipids are energetically much more stable within cell membranes where they are surrounded by phospholipids, than within micelles that are surrounded by water (Figure 2). Therefore, injection of micelles made of α-gal glycolipids into tumor lesions results in insertion of the α-gal glycolipids into the membranes of cells within the injected lesion and expression of α-gal epitopes on tumor cells, somewhat comparable to those naturally occurring on xenograft cells [52]. 

Extensive insertion of α-gal glycolipids into the cell membrane of human melanoma cells could be demonstrated after incubation of these cells with 1 mg/mL α-gal glycolipids for 2 h at 37 °C [53]. The multiple α-gal epitopes protruding from the tumor cells were found to readily bind the anti-Gal antibody, as shown by flow cytometry, whereas no binding of this antibody to melanoma cells was observed in the absence of α-gal glycolipids. Similar insertion was demonstrated *in vitro* with B16 mouse melanoma cells incubated with α-gal glycolipids and *in vivo* in B16 melanoma lesions injected with these glycolipids [52].

## 6. Outcomes of Intratumoral Injection of α-gal Glycolipids in Melanoma Lesions

Studies of intratumoral injection of α-gal glycolipids in the syngeneic model of KO mice bearing cutaneous B16 melanoma [52,53], suggested that interaction of anti-Gal with α-gal glycolipids will induce several processes that are also likely to occur in human melanoma lesions undergoing such treatment.

### 6.1. Extensive Recruitment of APC into the Treated Tumor Lesion

**Figure 3:**
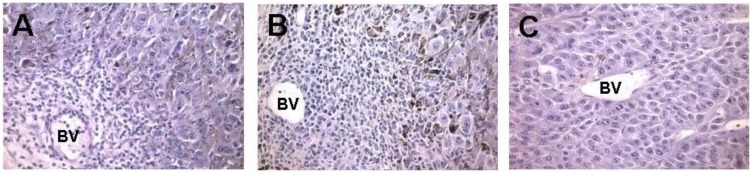
*In vivo* effect of injection of the α-gal glycolipids into B16 melanoma. Lesions reaching subcutaneous size of ~5 mm received one injection of α-gal glycolipids and were resected at different time points for histological analysis. (**A**) On day 2 post injection, mononuclear cells migrating from blood vessels (BV) are detected already; (**B**) On day 7 post injection, the infiltration is more extensive as indicated by the number of mononuclear cells migrating from blood vessels into the tumor; and (**C**) On day 7 post PBS injection, control tumors displayed no infiltration of mononuclear cells (×200).

Anti-Gal IgM and IgG molecules are released to the injection site from capillaries ruptured by the injecting needle. These anti-Gal molecules interact with α-gal epitopes on α-gal glycolipid micelles and rapidly induce a local complement cascade within the treated lesion. Complement cleavage chemotactic factors C5a and C3a, generated by this activation process, direct migration of APC such as monocytes/macrophages and dendritic cells into the injected tumor and induce intratumoral inflammation [52]. This could be demonstrated in B16 melanoma lesions (~5 mm in diameter) developing in KO mice within 5-6 days post subcutaneous inoculation of 1 × 10^6^ B16 cells. Such B16 melanoma lesions were injected with 1 mg α-gal glycolipids in 0.1 mL solution (~2 × 10^16^ α-gal epitopes/mg). Recruitment of APC is evident within two days post injection (Figure 3A). At that time point, multiple mononuclear cells are found in areas surrounding the blood vessels. Immunostaining and flow cytometry analysis of surface markers of the infiltrating cells indicated that a large proportion of these cells are macrophages and dendritic cells [52]. The infiltration of mononuclear cells increases by Day 7 post injection (Figure 3B). In melanoma lesions injected with PBS, no recruitment of APC is observed (Figure 3C), indicating that the migration of APC into lesions is directly associated with the presence of α-gal glycolipids within the injected tumors, rather than with the tissue damage caused by the injection needle. 

It should be stressed that a similar recruitment of APC into tumors can also be achieved by intratumoral injection of cytokines such as GM-CSF [11,61]. However, as indicated below, binding of anti-Gal to tumor cells further targets the tumor cells for uptake by APC via Fc/FcγR interaction. In contrast, cytokine mediated recruitment of APC to tumors does not contribute to the labeling of tumor cells for uptake by APC, thus, internalization of tumor cells by APC may be suboptimal. 

### 6.2. Destruction of Tumor Cells by Anti-Gal Binding to α-gal Glycolipids

The injected α-gal glycolipids insert spontaneously into the outer leaflet of the lipid bilayer of tumor cell membranes via the hydrophobic (lipophilic) ceramide tail (Figure 2). Binding of anti-Gal IgM to α-gal epitopes on the inserted α-gal glycolipids induces the destruction of the cells via complement dependent cytotoxicity (CDC), as in destruction of α-gal expressing xenograft cells [52]. Anti-Gal IgG bound to α-gal epitopes also facilitates antibody dependent cell cytotoxicity (ADCC) by macrophages and NK cells, following interaction of the Fc portion of anti-Gal on tumor cells with FcγR on these effector cells. These anti-Gal mediated cytolytic processes result in the prevention of additional growth of the injected tumor lesion and may result in regression of the injected tumor.

**Figure 4:**
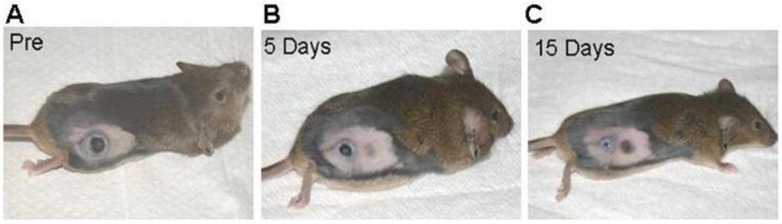
Effect of injected 1.0 mg α-gal glycolipids on tumor growth. (**A**) Pre-treated tumor; (**B**) Tumor on day 5 post α-gal glycolipids injection; (**C**) Tumor on day 15 post injection. Note the gradual regression of the tumor. The black spot developing in the skin near the regressing tumor was caused by a subcutaneous administration of α-gal glycolipids into normal skin, inducing local inflammation that results in transient activation of normal melanocytes.

Injection of α-gal glycolipids into B16 melanoma lesions resulted in stopping tumor growth in 65% of the treated KO mice, whereas in the remaining 35%, tumors continued to grow at a rate that was significantly slower than that of PBS injected tumors [52]. It should be stressed that B16 melanoma is a very aggressive tumor that usually doubles its size in KO mice (following subcutaneous implantation) every 4–8 days. In 20% of the tumors injected with α-gal glycolipids, the destruction of the tumor cells resulted in regression of the tumor. An example of tumor regression is shown in Figure 4 where the melanoma lesion and the adjacent normal skin were injected with α-gal glycolipids (1 mg in 0.1 mL). Injection of α-gal glycolipids into normal skin tissue resulted in a local inflammatory response due to anti-Gal/α-gal epitope interaction which induced temporary activation of melanocytes. This is seen as the dark spot appearing at the injection site by the treated lesion (Figure 4). As discussed in the safety section below, this activation of melanocytes in mice is transient and it always disappears within 3–4 weeks post injection (Figure 6).

### 6.3. Conversion of the Lesion into an Endogenous Vaccine by Anti-Gal Mediated Targeting of Tumor Cells to APC

**Figure 5:**
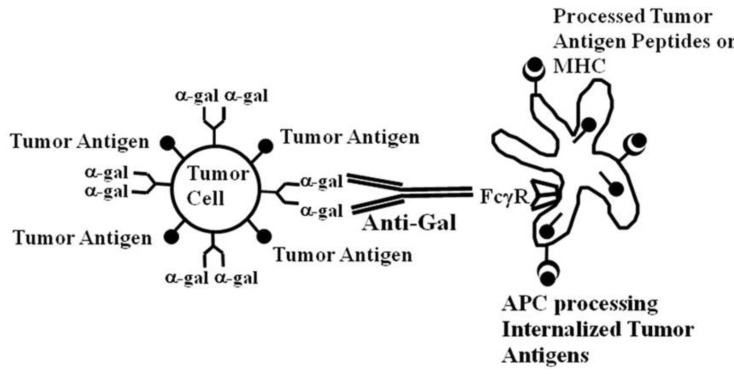
Anti-Gal mediated targeting of tumor cells to APC in lesions injected with α-gal glycolipids. Anti-Gal IgG binds *in situ* to α-gal epitopes on α-gal glycolipids inserted into tumor cell membranes. Subsequent interaction between the Fc portion of the bound anti-Gal and FcγR on the APC (schematic illustration of a dendritic cell) induces uptake of intact or lysed tumor cells by APC and thus, effective internalization of the tumor antigens (MAA in melanoma lesions). Internalized tumor antigens are processed and various immunogenic tumor antigen peptides (●) are presented by the APC in association with class I and class II MHC molecules. These immunogenic peptides can activate tumor specific T cells and elicit a protective anti-tumor immune response.

The destruction of melanoma lesions achieved by various ablation methods is likely to be more effective than the tumor destruction achieved by intratumoral injection of α-gal glycolipids (Figure 4). However, whereas the immune system may remain “oblivious” to MAA of ablated lesions, the intratumoral injection of α-gal glycolipids converts the treated lesion into an endogenous autologous tumor vaccine which elicits an immune response against autologous MAA. Anti-Gal IgG molecules bound to α-gal epitopes on glycolipids inserted into tumor cell membranes opsonize the melanoma cells and target them for effective uptake by APC, such as dendritic cells and macrophages (Figure 5). This uptake is the result of the interaction between the Fc portion of anti-Gal bound to the tumor cells and FcγR on APC [48,62]. As argued above, this anti-Gal mediated targeting of cells in injected lesions increases the immunogenicity of tumor antigens expressed in the internalized cells or cell membranes. 

By using B16 melanoma cells producing OVA (B16/OVA) as a surrogate MAA and analysis of SIINFEFKL (the immunodominant OVA peptide) expression on APC, it was possible to track the immunogenic tumor antigen peptides in KO mice. There was a marked increase in uptake, processing and presentation of OVA when the B16/OVA melanoma lesions were injected with α-gal glycolipids and the tumor cells were immunocomplexed with anti-Gal [52]. Moreover, analysis of the lymph nodes draining the treated tumor lesion indicated that the number of APC presenting SIINFEKL in tumors injected with α-gal glycolipids was much higher than that in lymph nodes draining tumors injected with PBS [52]. 

The increased uptake, processing, and presentation of MAA peptides by APC and their increased transport from the treated lesion to draining lymph nodes further resulted in increased activation of tumor specific T cells. This increased activation could be inferred from studies identifying tumor specific T cells in the spleens of treated KO mice [52,53]. In mice bearing B16/OVA tumor and treated by intratumoral injection of α-gal glycolipids, the number of SIINFEKL specific CD8+ T cells was found to be several fold higher than in mice receiving intratumoral injection of PBS. This analysis was performed both by ELISPOT measuring IFNγ secretion and by intracellular cytokine staining for IFNγ [52]. In addition, titer of anti-OVA antibodies was much higher in mice treated with α-gal glycolipids, implying an increased activation of Th cells that enable the activation OVA specific B cells [52]. 

The increased tumor specific T cell activation following intratumoral injection of α-gal glycolipids was further demonstrated with characteristic MAA in B16 bearing mice. Spleen cells analyzed by ELISPOT for IFNγ secretion, using immunodominant MAA peptides of tyrosinase and gp100, were found to include a much higher proportion of tumor specific T cells in tumor bearing mice treated with α-gal glycolipids than in mice with tumors injected with PBS [53]. 

## 7. Intratumoral Injection of α-gal Glycolipids Induces an Immune Response That Protects against Distant Metastases

The potential efficacy of immunotherapy with intratumoral injection of α-gal glycolipids in preventing the development of distant tumors could be determined by evaluating protection against contralateral tumor challenge. KO mice producing anti-Gal were inoculated with 1 × 10^6^ B16 cells in the right flank. Tumors reaching the size of 5 mm received three weekly injections of 1 mg α-gal glycolipids. One day after the third injection, the mice were inoculated in the left flank with 5 × 10^5^ B16 cells and subsequent tumor growth was monitored. Two-thirds of treated mice displayed no tumor growth in the left flank. In half of the remaining mice, the tumor growth in the left flank was significantly slower than growth of control PBS injected tumors [53]. This analysis was repeated in mice in which the primary tumor was ablated locally by intratumoral injection of ethanol. Although this ethanol treatment successfully destroyed the treated tumors, it did not elicit any protective immune response against challenge with B16 cells in the contralateral flank [53]. This control suggests that tumor cells killed by ablation do not elicit a protective immune response since they are not targeted effectively to APC.

A second approach for studying the ability of α-gal glycolipids to induce immune protection was to evaluate protection against a simulated distant established metastasis. This metastasis was generated by subcutaneous inoculation in the left flank with 10^4^ B16 cells at the same time that the right flank was inoculated with 10^6^ tumor cells. After 5–6 days, the tumor in the right flank was 5mm in diameter and ready to begin intratumoral treatment. Thus, by the time treatment was initiated, both right and left flank tumors had developed for 5–6 days without any treatment. At that time, all animals received two separate weekly intratumoral injections of either PBS or of α-gal glycolipids. In all control mice with right flank tumors injected with PBS, the left flank tumor developed into visible tumor of 4–6 mm [53]. However, in 50% of mice with right flank tumors injected with α-gal glycolipids, no lesions developed in the left flank during the 30 days of monitoring. In the remaining mice, the left flank tumor developed, but at a growth rate slower than the left flank tumor in the PBS treated mice [53]. These studies strongly suggest that intratumoral injection of α-gal glycolipids induces an immune response against autologous MAA so that it can destroy small pre-existing distant established tumors. This preclinical model may be analogous to the setting of a detectible local lesion and nondetectible distant micrometastases, as is the case in many high risk melanoma patients. 

## 8. Anti-tumor Protection in Mice Treated by Intratumoral Injection of α-gal Glycolipids Is Mediated Primarily by CD8+ T Cells

The identity of the cells mediating anti-tumor protection in treated mice could be determined by adoptive transfer studies with spleen lymphocytes. Naïve KO mice were inoculated subcutaneously with 5 × 10^5^ B16 cells. After 24 h, these mice received 40 × 10^6^ spleen lymphocytes from donors with their B16 tumors injected twice with α-gal glycolipids, or with PBS. The presence of the challenging tumor cells in the naïve KO mice prior to adoptive transfer simulates the presence of established micrometastases prior to immunotherapy. In the group receiving lymphocytes from donors with α-gal glycolipids injected tumors, ~70% of mice displayed no tumor growth and the remaining mice displayed slower tumor growth than in control mice [52,53]. In contrast, >75% of the mice receiving lymphocytes from donors with tumors treated with PBS developed large tumors within 30 days. No protection was observed in WT recipients of lymphocytes from WT donors with tumors injected with α-gal glycolipids [52]. WT mice can not produce anti-Gal because they are immunotolerant to α-gal epitopes, which are self antigens in these mice. Thus, in the absence of anti-Gal, no protective immune response could be elicited by intratumoral injection of α-gal glycolipids.

The protective effect of the lymphocytes transferred from KO mice with tumors injected with α-gal glycolipids was eliminated if these lymphocytes were depleted of CD8+ T cells [53]. Such depletion was achieved by the use of magnetic microbeads coated with anti-CD8 antibodies. These findings strongly suggest that transferred tumor specific CD8+ CTL are the primary cells that destroy the tumor cells in the naïve recipients. Interestingly, removal of CD4+ T cells from the lymphocytes transferred from a large proportion of donor mice with tumors injected with PBS resulted in increased protection against the tumor challenge [53]. These findings suggest that, in accord with previous reports [63], mice bearing B16 melanoma have CD4+ regulatory T (Treg) cells that inhibit the development of a protective anti-tumor immune response. The protection data with transferred lymphocytes from donors with α-gal glycolipids injected tumors further suggest that the elicited protective immune response is potent enough to overcome the suppressive effect of endogenous Treg in the tumor bearing mice [53]. 

## 9. Safety Studies with α-gal Glycolipids

Injected α-gal glycolipids were found to have no toxic effects on the treated mice. Five repeated intradermal injections of α-gal glycolipids resulted in no adverse effects in KO mice, no changes in CBC, in blood chemistry or in growth rate of the mice. Histological inspection of multiple organs, two months post two intradermal injections of 1 mg α-gal glycolipids (brain, spinal cord, heart, liver, lung, sciatic nerve, skin, liver, pancreas, testis, ovary, smooth muscle, cartilage, bone and bone marrow) revealed no infiltration of inflammatory cells to any of these organs and tissues. This implies that the insertion of α-gal glycolipids into normal cells in the mouse skin does not result in breakdown of immune tolerance to normal antigens, and thus, no autoimmune response is induced as a result of the treatment (unpublished observations). 

**Figure 6:**
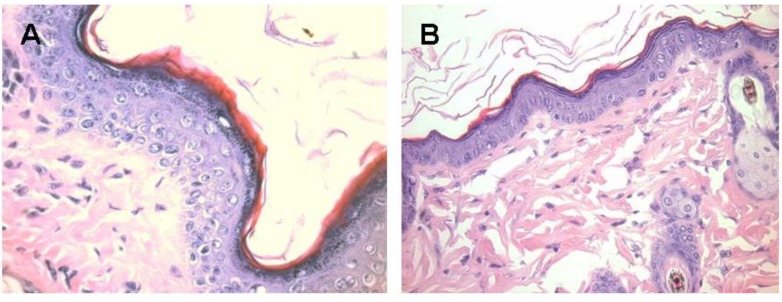
Melanocytes activation following subcutaneous injection of 1.0 mg α-gal glycolipids into the KO mouse. **A.** The epidermis displays 4–5 layers of cells 7 days post injection in the black spot area. The apical region under the keratinous layer (stained in red) is filled with many melanin granules. **B.** The injected skin four weeks post injection. The epidermis displays normal structure of ~2 layers of epidermal cells, the amount of melanin granules is residual and the overall color of the skin returns to normal pink (x400).

In 60% of the KO mice, intradermal injection of α-gal glycolipids resulted in a local inflammatory response that stimulated melanocytes to produce more melanin in the form of a black spot which developed in the normal skin injection site within 4–7 days (Figure 4, Figure 6). Such a black spot completely disappears within 21–28 days post injection due to turnover of the normal skin epidermis.

Safety studies on α-gal glycolipids further addressed the question of the potential effects of α-gal glycolipids in the circulation, if they reach the blood from the injection site. For this purpose, anti-Gal producing KO mice were injected intravenously into the tail vein with five weekly injections of 1 mg α-gal glycolipids. Subsequently, the mice were monitored for 30 days. No adverse effects were detected in these mice at any time point (unpublished observations). These studies suggest that anti-Gal/α-gal glycolipids interaction in the blood results in no adverse effects. 

Several observations in humans suggest that α-gal glycolipids may not have adverse effects in patients that received them. Glutaraldehyde fixed porcine heart valves express an abundance of α-gal epitopes that are not affected by the fixation and which readily interact with the anti-Gal antibody (unpublished observations). These valves are widely used to replace diseased heart valves, without apparent detrimental effects. Administration of live cells expressing α-gal epitopes into humans also does not seem to be associated with any adverse effect. This was observed in patients with diabetes who received transplants of 3–6 gm live fetal pig islet cells, which express large amounts of α-gal epitopes [64]. Moreover, the α-gal epitope is abundantly present in cow and pork meat, ingested routinely without demonstrated immune toxicity.

## 10. Planned Studies of Immunotherapy for Melanoma Patients with α-gal Glycolipids

Based on the efficacy and safety studies in KO mice, the FDA has approved a Phase I clinical trial for studying intratumoral injection of α-gal glycolipids in patients with advanced solid tumors and patients with advanced melanoma (IND-12946). The study includes two protocols.

The first protocol is a Phase I study for patients with a variety of advanced cancers that have measurable tumor sites that are not readily accessible for cutaneous or subcutaneous injection. These patients receive a single injection of α-gal glycolipid into their internal solid tumors. The administration of α-gal glycolipids is by colonoscopy, endoscopy, laparoscopy, or ultrasound guidance, depending on the site of the tumor. Using standard Phase I dose escalation, each subsequent group of patients receive increasing doses of α-gal glycolipids. This study is performed in the Department of Surgery, UMass Medical School (Dr. Giles Whalen, clinical PI). The doses used for the various cohorts are 0.1, 1.0, 10 and 100 mg α-gal glycolipids per injection. So far, patients were treated with the doses of 0.1, 1.0 and 10 mg α-gal glycolipids and were found to have no indication of toxicity following this treatment.

The second protocol under this IND includes the study of α-gal glycolipids treatment in patients with advanced melanoma who have at least one readily injectable cutaneous or subcutaneous lesion. In contrast to the first protocol, this protocol includes two injections of α-gal glycolipids, over a four week interval. The doses are as in the first protocol. The second injection (the same dose as the first) is administered in order to maximize immunogenicity of the treated lesion. While toxicity is not anticipated, to maximize safety patients will receive a small intradermal “test dose” injection of α-gal glycolipids prior to the second treatment of α-gal glycolipids and then be observed for one hour for evidence of allergic reaction before proceeding with the second injection. This protocol plans to evaluate the tumors that receive intratumoral α-gal glycolipids as well as tumors distant to the injected one in these same patients. The injected lesions will be biopsied and analyzed for an induced inflammatory response. In addition, anti-tumor activity at a site distant from the injected index lesion will be evaluated with tumor biopsies in patients with additional readily biopsiable lesions. Immune monitoring assays of peripheral blood mononuclear cells will also be included to determine T cell responses to autologous tumor and/or to common MAA. The study is performed in the melanoma clinic at the Carbone Cancer Center of the University of Wisconsin in Madison, WI (Dr. Mark Albertini clinical PI) and in the Department of Surgery, UMass Medical School (Dr. Giles Whalen, clinical PI).

We anticipate that determination of the maximum tolerated dose (MTD) of α-gal glycolipids injected into melanoma lesions will be followed by Phase II studies in which these glycolipids will be injected into several lesions at a total amount equivalent to the MTD. The efficacy of the treatment in eradicating the injected lesions and inducing immune mediated shrinkage (clinical measurement) or necrosis (determined by histological review of biopsied tumors) of distant metastases will be determined. The results of this treatment are likely to vary from one patient to the other and will depend on the number and immunogenicity of MAA in the individual patient and on the potency of the immune system in the treated patient. In a proportion of the patients, the combination of these factors may result in the generation of an immune response against the autologous MAA that destroys tumor cells in micrometastases. 

It is not clear at present whether the proposed treatment can elicit an immune response potent enough to destroy visible metastases. Based on the preclinical data in our mouse model, we hypothesize that the systemic anti-MAA immune response induced by direct injection of α-gal glycolipids into measurable tumors will be more effective in destroying undetectable micrometastases than in eradicating distant measurable macrometastases. For many patients, detectable metastases can be destroyed by ablation or resection. Moreover, even measurable metastases that are injected with α-gal glycolipids can be destroyed by standard treatment of ablation or resection in case the anti-Gal response against the tumor cells with inserted α-gal glycolipids does not suffice for the destruction of the whole lesion. Thus, we envision a future approach for high-risk patients that combines injection of α-gal glycolipids into readily injectable measurable lesions, coupled with subsequent (several weeks after the intratumoral injection of α-gal glycolipids) resection or standard ablation of uninjectable lesions and injected lesions that have not resolved from the injection of α-gal glycolipids. This combined approach may allow a sufficient period for the “education” of the immune system to recognize autologous MAA and destroy micrometastatic cells expressing these MAA. The goal would be using the combination approach to eliminate all visible lesions, thereby putting the patient into clinical remission, while inducing an effective immune response that destroys non-detectable micrometastases. Ideally, this approach would also prevent the progression of these micrometastases into recurrent lethal macroscopic melanoma metastases. 

Several years of testing may be required to clinically develop this approach. Based on the efficacy in the preclinical tumor-bearing mouse model and the potency of the anti-Gal antibody response in rejecting organ xenografts, we feel that this concept merits the ongoing and proposed clinical testing.

## Abbreviations

α-gal epitope: Galα1-3Galβ1-4GlcNAc-R epitope;
α1,3GT: α1,3-galactosyltransferase;
B16: mouse melanoma cells lacking α-gal epitopes;
CPH: ceramide pentahexoside;
CTL: cytotoxic T cells;
FcγR: Fcγ receptor;
KO: knockout mice for α1,3GT;
MAA: melanoma associated antigens;
OVA: ovalbumin;
RabRBC: rabbit RBC;
TAA: tumor associated antigens;
WT: wild type mice producing α-gal epitopes.
